# Early Life Glucocorticoid Exposure Modulates Immune Function in Zebrafish (*Danio rerio*) Larvae

**DOI:** 10.3389/fimmu.2020.00727

**Published:** 2020-04-29

**Authors:** Ruud van den Bos, Suzanne Cromwijk, Katharina Tschigg, Joep Althuizen, Jan Zethof, Robert Whelan, Gert Flik, Marcel Schaaf

**Affiliations:** ^1^Department of Animal Ecology and Physiology, Institute of Water and Wetland Research, Faculty of Science, Radboud University, Nijmegen, Netherlands; ^2^Animal Sciences and Health Cluster, Institute of Biology, Leiden University, Leiden, Netherlands

**Keywords:** cortisol, glucocorticoid receptor, zebrafish, larvae, tail fin regeneration, lipopolysaccharide

## Abstract

In this study we have assessed the effects of increased cortisol levels during early embryonic development on immune function in zebrafish (*Danio rerio*) larvae. Fertilized eggs were exposed to either a cortisol-containing, a dexamethasone-containing (to stimulate the glucocorticoid receptor selectively) or a control medium for 6 h post-fertilization (0–6 hpf). First, we measured baseline expression of a number of immune-related genes (*socs3a, mpeg1.1, mpeg1.2*, and *irg1l*) 5 days post-fertilization (dpf) in larvae of the AB and TL strain to assess the effectiveness of our exposure procedure and potential strain differences. Cortisol and dexamethasone strongly up-regulated baseline expression of these genes independent of strain. The next series of experiments were therefore carried out in larvae of the AB strain only. We measured neutrophil/macrophage recruitment following tail fin amputation (performed at 3 dpf) and phenotypical changes as well as survival following LPS-induced sepsis (150 μg/ml; 4–5 dpf). Dexamethasone, but not cortisol, exposure at 0–6 hpf enhanced neutrophil recruitment 4 h post tail fin amputation. Cortisol and dexamethasone exposure at 0–6 hpf led to a milder phenotype (e.g., less tail fin damage) and enhanced survival following LPS challenge compared to control exposure. Gene-expression analysis showed accompanying differences in transcript abundance of *tlr4bb, cxcr4a, myd88, il1*β*, and il10*. These data show that early-life exposure to cortisol, which may be considered to be a model or proxy of maternal stress, induces an adaptive response to immune challenges, which seems mediated via the glucocorticoid receptor.

## Introduction

In teleosts, like zebrafsh (*Danio rerio)*, cortisol is the main endogenous corticosteroid, which is secreted when individuals perceive situations as stressful (1). Like in other vertebrate species, cortisol binds in teleosts to the mineralocorticoid receptor (MR) and the glucocorticoid receptor (GR), which affects the transcription rates of genes following ligand binding (2–4). Since the GR has a lower affinity for cortisol than the MR, the GR mediates the actions of cortisol during stress, which involves optimizing energy expenditure by tuning the balance between and within physiological systems, like an organism's metabolism and its immune, cardiovascular and central nervous system [e.g., (1, 4, 5)]. Following long-term exposure to stress, baseline levels of cortisol are increased, reflecting the allostatic load that the environment imposes on an organism (5, 6). Cortisol has been shown to signal through the GR already during the very early stages of embryonic development; in oocytes maternally deposited cortisol and GR mRNA are present (7–9). These cortisol levels may reflect the allostatic load that the mothers experience in their environment and it has therefore been hypothesized that these deposited cortisol levels are important for preparing the offspring for the expected allostatic load that larvae will encounter in the prevailing environment, thereby programming their cortisol secretion and the functioning of physiological systems to meet expected demands (4, 10). While in previous studies we focussed on the effects of cortisol exposure [between 0 and 6 h post fertilization (hpf)] on vigilance-related behavior, baseline cortisol levels and metabolism in larvae (10, 11) in the present study we focussed on the functioning of the immune system, in particular the innate immune system on which zebrafish larvae rely (12). Cortisol exposure may be considered to be a model or proxy of maternal stress (10, 13–15).

Zebrafish is a highly suitable animal model to study early life events in the fields of biomedical research, behavioral biology and eco-toxicology [e.g., (4, 16–19)]. Fertilized eggs develop into independently feeding larvae outside the mother, without parental care, and can easily be maintained under different experimental conditions as well as pharmacologically manipulated. In zebrafish, it has been demonstrated that mothers deposit cortisol and GR mRNA in oocytes (7–9). These cortisol levels decrease over the first 24 h post fertilization, after which zygotes gradually start to produce cortisol by the then developing interrenal cells (7–9, 20). After hatching (48–72 hpf) pituitary control over interrenal cortisol production starts and it takes another 4–5 days before the hypothalamus-pituitary-interrenal (HPI) axis is fully functionally mature (2, 3). Maternal GR mRNA is present during the first 6 hpf, and at 8–9 hpf zygotic expression of the GR commences, while the MR mRNA production starts at 24 hpf (3, 7, 8).

In several studies the effect of cortisol exposure during early embryonic stages has been investigated in zebrafish. In these studies cortisol levels were increased by injection of cortisol into the yolk of one-cell stage embryos [e.g., (13–15)] or through addition of cortisol to the medium (10, 21). These studies showed that, as a result of the cortisol exposure during early embryonic stages, larval baseline levels of cortisol were increased (10, 13, 14, 21). For example, in a recent study, we have demonstrated that cortisol exposure between 0 and 6 hpf increased baseline cortisol levels 5 days post fertilization (dpf) of larvae from the AB strain (10).

Exposure to cortisol (0–5 dpf) has been shown to lead to an enhanced expression of immune-related genes in zebrafish larvae at 5 dpf (21), suggesting that early cortisol exposure increases the activity of the immune system. In the present study, we have first measured the expression of a selected number of these up-regulated genes at 5 dpf (*socs3a, mpeg1.1, mpeg1.2, irg1l*) following our 0–6 hpf exposure to cortisol (10) in zebrafish larvae of the AB strain to assess whether our method produces similar effects. Tüpfel long-fin (TL) is another widely used zebrafish strain next to AB and is characterized by spots rather than stripes as well as long fins rather than short fins. In previous studies we have observed consistent differences between larvae of the AB strain and larvae of the TL strain at the level of both HPI-axis activity and behavior (10, 22–24). We have attributed these differences to the mutation in the connexin 41.8 gene that leads to spots [for discussion: see (24)]. Measuring the expression of these genes in larvae of the TL strain next to larvae in the AB strain may therefore reveal how robust our findings are. Finally, to assess the role of the GR in more detail, we exposed fertilized eggs 0–6 hpf to dexamethasone, a specific GR agonist (25).

To functionally assess the activity of the immune system following early life exposure to cortisol or dexamethasone, we used two experimental models for immune activation. First, we used the tail fin amputation assay. This is a well-established model in which amputation of the tail triggers expression of many pro-inflammatory molecules and the recruitment of innate immune cells (neutrophils and macrophages) toward the wounded area (26–28). The tail fin amputation assay was performed using the double transgenic fish line *Tg(mpx:GFP/mpeg1:mCherry-F)* (27, 29). Recruitment of neutrophils and macrophages was determined following tail fin amputation in larvae at 3 dpf (30, 31). Second, we used a sepsis model, which involved a challenge with lipopolysaccharide (LPS), the membrane component of Gram-negative bacteria, in 4 dpf larvae. We measured survival, phenotypical changes, and the expression of a series of LPS-responsive genes (32–35).

## Materials and Methods

### Subjects, Spawning, and Care

Housing conditions and breeding procedures were similar as those reported in Van den Bos et al. (10, 22, 23), AB and Tüpfel long-fin (TL) strains, or Xie et al. (31), the double transgenic fish line *Tg(mpx:GFP/mpeg1:mCherry-F)*. They were kept in recirculation systems (~28°C) under a 14:10 h light-dark cycle and fed twice daily.

Breeding started at least 1 h after the last feeding of zebrafish (>16:00 h). Males and females of the AB or TL strain were placed in a zebrafish breeding tank, separated by a partitioning wall, with water of ~28°C. After turning on the lights the next morning, the partitioning wall was removed and tanks were placed at a slight angle, such that the fish had the possibility to move into shallow water to spawn.

### Cortisol/Dexamethasone Exposure During Early Embryonic Development (0–6 hpf)

Cortisol (hydrocortisone; Sigma-Aldrich, Zwijndrecht, the Netherlands) and dexamethasone (Sigma-Aldrich, Zwijndrecht, the Netherlands) were dissolved in 96% ethanol in the required stock solution concentrations and stored at −20°C. From these stock solutions media with the appropriate concentration were freshly prepared for each experiment (10, 36): cortisol-containing medium: 400 μg/l cortisol (1.1 μM), 0.4 ml/l 96% ethanol (0.04% v/v), 5 mM NaCl, 0.17 mM KCl, 0.33 mM CaCl_2_, 0.33 mM MgSO_4_ in dH2O; dexamethasone-containing medium: 430 μg/l dexamethasone (1.1 μM), 0.4 ml/l 96% ethanol [or 1 ml/l 96% ethanol (0.1% v/v); depending on the specific experiment], 5 mM NaCl, 0.17 mM KCl, 0.33 mM CaCl_2_, 0.33 mM MgSO_4_ in dH2O. Control medium consisted of: 0.4 ml/l 96% ethanol (or 1 ml/l 96% ethanol; depending on the specific experiment), 5 mM NaCl, 0.17 mM KCl, 0.33 mM CaCl_2_, 0.33 mM MgSO_4_ in dH2O.

For each experiment we used between 8 and 12 breeding trios (one female and two males). Directly following each spawning and fertilization, eggs were collected and randomly assigned to Petri dishes filled with either cortisol-containing medium, dexamethasone-containing medium or control medium. Thus, for each treatment we had multiple Petri dishes with eggs from all breeding pairs. Within 1–1.5 hpf Petri dishes were placed in an incubator set at 28.5°C (300–350 lux). Eggs were exposed to these solutions for 6 hrs. It has been shown that both cortisol and dexamethasone diffuse inside the eggs in this period (37). In addition, we have already shown in earlier studies that this procedure is effective in eliciting changes in physiology and behavior at 3–5 dpf (10, 36).

Following this, cortisol-containing, dexamethasone-containing and control media were replaced by E3 medium (5 mM NaCl, 0.17 mM KCl, 0.33 mM CaCl_2_, 0.33 mM MgSO_4_, 3 ml/l 0.01% (w/v) methylene blue in dH2O). Embryos were rinsed twice in E3 medium to ensure that the original treatment media were completely removed. Petri dishes were returned to the incubator allowing the embryos to develop further (28.5°C; 14:10 h light-dark period (lights on: 09.00–23.00 h); light phase: 300–350 lux; dark phase; 0 lux). At 1 and 4 dpf E3 medium was refreshed and unfertilized eggs, dead eggs/embryos/larvae and chorions were removed from the dishes.

All experiments were carried out in accordance with the Dutch Experiments on Animals Act (http://wetten.overheid.nl/BWBR0003081/2014-12-18), the European guidelines for animal experiments (Directive 2010/63/EU; http://eur-lex.europa.eu/legal-content/NL/TXT/HTML/?uri=CELEX:32010L0063) and institutional regulations (Radboud University or Leiden University). Larvae were euthanized by placing them in ice slurry for at least 20 min followed by adding bleach to the slurry. In case of anesthesia 0.01% 2-phenoxyethanol (Sigma Aldrich, Zwijndrecht, the Netherlands; experiments Radboud University) or 0.02% buffered aminobenzoic acid ethyl ester (tricaine; Sigma Aldrich, Zwijndrecht, the Netherlands; experiments Leiden University) was used.

### Baseline Gene Expression Analysis

**Figure 1:**
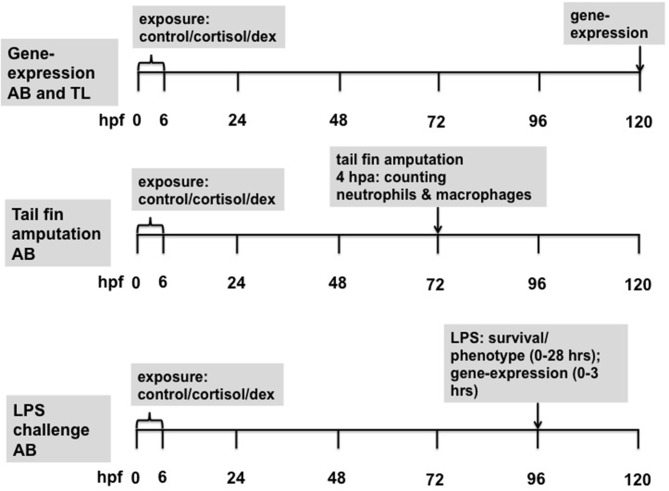
Experimental time-line of the different experiments. dex, dexamethasone.

Following 0–6 hpf to the different media, larvae of the AB and TL strain were sampled for gene expression at 5 dpf between 16.00 and 19.00 h in line with our earlier study [(10); experimental time-line Figure 1]. Genes of interest were: *socs3a, mpeg1.1, mpeg1.2*, and *irg1l* [for primer sequences of these genes: see (22)]. Gene expression was determined by qPCR analysis as described below.

### Tail Fin Amputation Assay

The tail fin amputation assay was performed using the double transgenic fish line *Tg [mpx:GFP/mpeg1:mCherry-F;* (27, 29)]. Three-day-old zebrafish larvae were anesthetized in E3 medium containing 0.02% buffered tricaine and loaded onto 2% agarose-coated Petri dishes (experimental time-line: Figure 1). Amputation was performed with a 1 mm sapphire blade (World Precision Instruments, Friedberg, Germany) using a Leica M165C stereomicroscope and a micromanipulator (Leica Microsystems BV, Amsterdam, the Netherlands; see below, **Figure 3**). Larvae were fixed in 4% paraformaldehyde in PBS at 4 h post amputation (hpa) and stored overnight at 4°C. The following day larvae were washed twice for 1 min and then twice for 5 min in PBS containing 0.01% Tween 20 (Sigma Aldrich, Zwijndrecht, the Netherlands).

A Leica M205FA fluorescence stereomicroscope supported by LAS software (version 4.12.0; Leica Microsystems BV, Amsterdam, the Netherlands) was utilized to visualize the leukocytes. Detection of neutrophils and macrophages was based on their fluorescent GFP and mCherry signals, respectively. To quantify cell migration toward the wounded area, cells within a distance of 200 μm from the amputation site were counted manually, as previously described (31). Data (numbers of migrated neutrophils and macrophages per individual) were pooled from three individual experiments (*n* > 10 per experiment), and the presented data are means (± SEM).

### LPS Exposure

First, we conducted a pilot study to assess the optimal LPS dose, exposure duration and parameters to be measured [protocols adapted from: (32–35)]. Incubation for 30 min in 150 μg/ml LPS (B11.04; Sigma Aldrich, Zwijndrecht, the Netherlands) was effective in eliciting a robust increase in *il1*β expression (assessed using qPCR analysis), changes in tail fin morphology (swollen or damaged tails) and increased levels of reactive oxygen species [ROS; measured by a fluorescent labeling method according to (35)]. Hence, we used this dose in subsequent experiments.

Subsequently, two experiments were conducted. In both experiments 4 dpf larvae were exposed to 150 μg/ml LPS (B11.04; Sigma Aldrich, Zwijndrecht, the Netherlands) in E2 medium or control E2 medium for 30 min in Petri dishes (*n* = 50 in 25 ml; experimental time-line: Figure 1). Following this exposure, LPS-containing medium or control E2 medium was replaced by fresh E2 medium (larvae were rinsed two times to ensure that the original media were removed). Larvae either remained in the Petri dishes for sampling for gene expression at later time points or were transferred individually to 24 wells plates (Greiner Bio-One BV, Alphen a/d Rijn, the Netherlands) for assessing phenotypical changes and survival (volume per well: 1–1.5 ml). Six treatment groups were thus created: 0–6 hpf control, cortisol or dexamethasone treatment, combined with either 4 dpf LPS or control treatment (for 30 min).

In the first experiment the level of gene expression at 0, 0.5 h (i.e., directly following exposure), 1 hr (i.e., 30 min after ending exposure) and 3 h [i.e., 2.5 h after ending exposure; (34)] was determined by qPCR analysis as described below. Genes of interest [see papers by: (33–35, 38)] were genes encoding proteins involved in barrier function of the vascular endothelium (*cldn5a, cldn2, oclnb*), Toll-like receptors (*tlr2, tlr4ba, tlr4bb, tlr5a, tlr5b*), and regulators of the immune response (*il1*β, *il10, myd88, cxcr4a, cxcr4b, ptpn6*). Primer sequences are listed in Table 1.

**Table 1:** Nucleotide sequences of forward and reverse primers used for qPCR.

**Gene**	**Forward primer sequence (5^**′**^-3^**′**^)**	**Reverse primer sequence (5^**′**^-3^**′**^)**	**Amplicon length (bp)**	**Source**
*elf1a*	CTGGAGGCCAGCTCAAACAT	TCAAGAAGAGTAGTACCGCTAGCATTAC	85	NM_131263.1
*rpl13*	TCTGGAGGACTGTAAGAGGTATGC	AGACGCACAATCTTGAGAGCAG	147	NM_212784
*cldn5a*	CTCGGGGGAAGCATACTCTG	TATTCCCCGTTGGATGTGGC	101	NM_213274.1
*cldn2*	CCCCTACTGGGCAACATCTG	CTTGCAGGTCGACGGTTAGT	155	XM_005170272.4
*Oclnb*	TGACAAAGTCAGTGCTGCTCA	GGATATTCATCTGTGTCAAGCTCAT	162	NM_001008618.1
*tlr2*	TGCTGTCGGTCGATTACCTG	ACACAGGGAAAACGAAGGCT	130	NM_212812.1
*tlr4ba*	ACTTTCAAGATTTCCGTTGAGGTAT	CCACAAGAACAAGCCTTTGCAG	209	NM_001131051.1
*tlr4bb*	ATCACACTGCTGTGTTGCGA	AGGCCAGTGAGAGCTTTGTG	178	NM_212813.2
*tlr5a*	TGGTTCCGGTTGTGAGTTCC	TCTTGAAGATGACACCGCGT	162	XM_001919017.6
*tlr5b*	GGTCTTGAAGCGAGTGGTGT	CATTTTGCGCCAAGGTCAGT	118	NM_001130595.2
*il1β*	GATGGCATGCGGGCAATATG	AGCTCATTGCAAGCGGATCT	116	NM_212844.2
*il10*	GACCATTCTGCCAACAGCTC	ACCATATCCCGCTTGAGTTCC	102	NM_001020785.2
*myd88*	GTTTGCGCTCAGTCTTTGCC	GGTCAGAAAGCGCAGAATGC	103	NM_212814.2
*cxcr4a*	ACTTGTACAGCAGCGTCCTC	CTTTGGCGAACACCAAGTCG	174	NM_131882.3
*cxcr4b*	GCGCCTTTTTGAGCACACTT	ATTGCTGACTGAGAGGTCGC	127	NM_131834.1
*ptpn6*	ACAGGAAGAACTGCGCTCAT	TGCAGCACAGCCAAGTAGAT	225	NM_199960.1

*Reference genes: elongation factor 1α (elf1α); ribosomal protein L13 (rpl13)*.

In addition, in the first experiment survival and phenotypical changes were determined at 0, 4 (i.e., 3.5 h after ending exposure) and 28 h (i.e., 27.5 h after ending exposure). Phenotypical changes included [see (35)]: changes in tail fin morphology (normal, swollen (oedema) or damaged), presence of heart oedema and changes in shape (straight or curved). In the second experiment (that also served as replicate for the first experiment) survival and phenotypical changes were measured at 0, 0.5, 1, 3, 6, and 24 h (i.e., before exposure, directly after exposure, 0.5 h after ending exposure, 2.5 h after ending exposure, 5.5 h after ending exposure and 23.5 h after ending exposure).

### Gene Expression Analysis

For the assessment of gene expression levels by qPCR analysis, 3–5 larvae were transferred to a 2-ml Eppendorf tube; thus, one sample contained material from 3 to 5 larvae. Residual medium was removed with a pipette, tubes were snap frozen in liquid nitrogen, kept on ice during the sampling procedure, and subsequently stored at −80°C until total RNA extraction.

RNA isolation, RNA preparation, removal of genomic DNA from the samples and synthesis of cDNA was performed according to the protocol described in Van den Bos et al. (22). Total RNA content of each sample was isolated. This was done by homogenizing the tissue with 400 μl Trizol reagent (Invitrogen, Carlsbad, USA) in a Grinding Mill (Retsch GmbH, Germany) for 20 s at 20 Hz. After homogenisation, samples were kept at room temperature for 5 min. Next, 80 μl chloroform was added and the solution was mixed by shaking for 15 s. Afterwards, samples were kept at room temperature for 2 min. The samples were centrifuged at 14,000 rpm for 10 min in a cooled centrifuge (4°C) and the aqueous phase of the samples was transferred to a new tube. To this phase, 200 μl isopropanol was added and this solution was mixed well by inversion of the tube. The solution was then stored at−20°C for 2 h and centrifuged afterwards for 15 min at 14,000 rpm in a cooled centrifuge (4°C). The supernatant was decanted and the pellet washed with 500 μl 75% ethanol and centrifuged 10 min at 14,000 rpm in a cooled centrifuge (4°C). The supernatant was decanted, after which the pellet was centrifuged for 5 s to remove all the remaining supernatant using a pipette. The pellet containing the RNA was air-dried for 10 min at room temperature and afterwards dissolved in 100 μl ice cold DEPC-treated dH2O. To this RNA solution, 10 μl 3M NaAc (pH 5.4) and 250 μl 100% ethanol were added. The solution was mixed by inverting the tube and samples were stored for 2 h at−20°C. Subsequently, the samples were centrifuged for 15 min at 14,000 rpm in a cooled centrifuge (4°C), and the supernatant was decanted and the pellet washed as described earlier. Finally, the RNA pellet was dissolved in 15 μl DEPC-treated dH2O. The concentration and quality of RNA in each sample were measured using a nanodrop spectrophotometer at 260 nm wavelength (Nanodrop, Wilmington, DE, USA).

Isolated RNA was treated with DNase to remove any (genomic) DNA from the sample; 400 ng RNA was transferred into a PCR strip, and DEPC-treated dH2O was added to a volume of 8 μl. To this, 2 μl of DNase mix was added, containing 1 μl 10x DNase I reaction buffer and 1 μl (1 U/μl) amplification grade DNase I (both from Invitrogen, Carlsbad, USA). The resulting mix was incubated for 15 min at room temperature. Afterwards, 1 μl 25 mM EDTA was added to stop the DNase reaction and the reaction mix was incubated for 10 min at 65°C and returned on ice.

After the DNase treatment, samples were used to synthesize cDNA by the addition of 1 μl random primers (250 ng/μl), 1 μl 10 mM dNTP mix, 4 μl 5 × 1st strand buffer, 1 μl 0.1M DTT, 1 μl RNase inhibitor (10 U/μl), 0.5 μl Superscript II (reverse transcriptase) (200 U/μl) (all from Invitrogen, Carlsbad, USA) and 0.5 μl DEPC-treated dH2O. The resulting mix was incubated for 10 min at 25°C for annealing of the primers and then 50 min at 42°C for reverse transcription. Enzymes were hereafter inactivated by incubating samples at 70°C for 15 min. Finally, 80 μl dH2O was added to dilute the samples five times for the qPCR reaction.

To measure the relative gene expression in each sample, real-time qPCR was carried out for each gene of interest. For each qPCR reaction, 16 μl PCR mix (containing 10 μl SYBR green mix (2x) (BioRad, Hercules, USA), 0.6 μl forward and reverse gene-specific primer (10 μM) and 4.8 μl H2O) was added to 4 μl of cDNA. The qPCR reaction (3 min 95°C, 40 cycles of 15 s 95°C and 1 min 60°C) was carried out using a CFX 96 (BioRad, Hercules, USA) qPCR machine. Analysis of the data was carried out using a normalization index of two reference genes [viz. *elongation factor alpha* (*elf1a*) and *ribosomal protein L13* (*rpl13*)] (39).

### Statistics

For gene expression analyses, outliers were removed following Grubb's outlier test (*p* ≤ 0.01). We explored the interrelationships of transcript abundance levels using Principal Component Analysis (PCA) with orthogonal rotation [Varimax rotation with Kaiser normalization; see (23)]. In case of missing samples, data were excluded list-wise. The number of retained components was based on eigenvalues (>1) and visual inspection of the scree plot. The Kaiser- Meyer-Olkin (KMO) measure of sampling adequacy and Bartlett's test of sphericity were done to ensure that data obeyed analysis criteria; both are measures to assess whether the correlation matrix is suited for factor analysis (40). Component scores were saved and used for further statistical analysis. The following component loading cut-off points were considered: ≤-0.600 or ≥0.600 (40, 41).

For the basal gene expression values a two-way or three-way Analysis of Variance (ANOVA) was run with treatment, strain or batch (where applicable) as independent factors. In the tail fin amputation assay a Student's *t*-test was run on the number of neutrophils or macrophages at 4 hpa comparing 0–6 hpf treatment groups (cortisol vs. control; dexamethasone vs. control).

In the LPS exposure experiment, for gene expression a multivariate analysis of variance (MANOVA) was run (to account for multiple comparisons) followed by univariate analysis of variance [0–6 hpf treatment and time as independent factors followed by *post-hoc* testing (Tukey HSD)]. In addition per time point a one-way ANOVA was run with 0–6 hpf treatment as factor followed by *post-hoc* testing (Tukey HSD).

In the two LPS exposure experiments differences in survival rate were assessed using the Kaplan-Meijer procedure (Log Rank Mantel-Cox). Differences in phenotypical changes were compared using Chi-square tests.

Significance was set at *p* ≤ 0.05 and trends are indicated (*p* ≤ 0.10) where appropriate; ns: not significant: *p* > 0.10. Unless otherwise stated all *p*-values are two-tailed. All statistical analyses were done using IBM SPSS version 23 (IBM, Armonk, NY, USA).

## Results

### Baseline Gene Expression Analysis

Rather than analyzing transcript abundance of different genes following glucocorticoid treatment (cortisol or dexamethasone, 0–6 hpf) separately, we explored the effects of exposure through the interrelationships of transcript abundance of genes using PCA.

**Figure 2:**
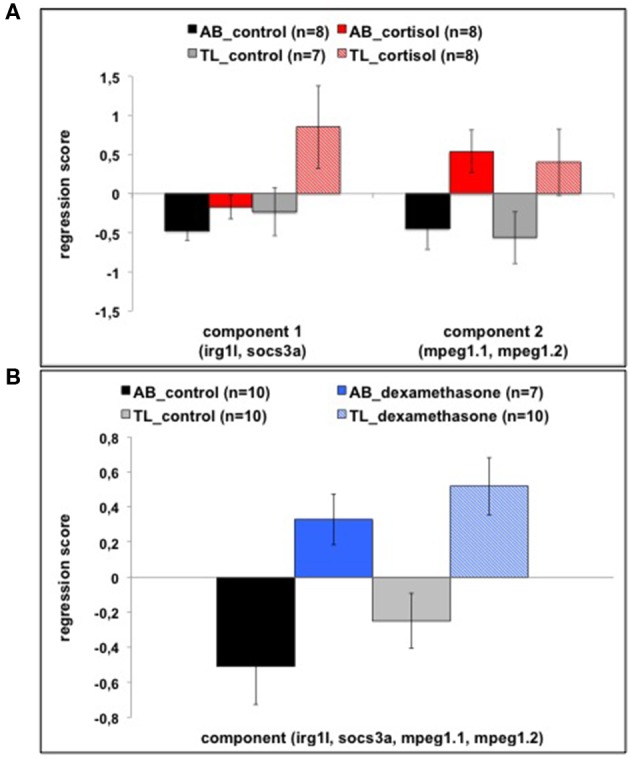
**(A)** Regression scores (mean ± SEM) of components 1 and 2 from the PCA for the different treatments (cortisol or control) and strains (AB or TL). Genes that contributed to the components are indicated in the figure. One subject was removed from the statistical analyses (TL control) as it was a consistent outlier following Grubb's outlier test. **(B)** Regression scores (mean ± SEM) of the only component of the PCA for the different treatments (dexamethasone or control) and strains (AB or TL). Genes that contributed to the component are indicated in the figure.

A PCA for the 0–6 hpf cortisol treatment experiment revealed two components (Figure 2A; Supplementary Figure 1A shows the transcript abundance of individual genes). The KMO value was sufficiently high (0.522) and Bartlett's test of sphericity was highly significant (Chi-square = 17.707, df = 6, *p* ≤ 0.007), indicating that the data were adequate for a PCA. The first component (explaining 44.0% of the variance) was comprised of *irg1l (*loading: 0.854) and *socs3a* (loading: 0.890); the second component (explaining 28.9% of the variance) of *mpeg1.1* (loading: 0.890) and *mpeg1.2* (loading: 0.890). Each component was analyzed separately using ANOVA. Treatment (0–6 hpf) with cortisol enhanced transcript abundance of genes in component 1 (*irg1l* and *socs3a*) independent of strain [three-way ANOVA (strain, treatment, batch): treatment: *F*_(1, 23)_ = 6.477, *p* ≤ 0.018]. Transcript abundance was overall higher in TL than AB larvae [strain: *F*_(1, 23)_ = 7.023, *p* ≤ 0.014]. Treatment (0–6 hpf) with cortisol enhanced transcript abundance of genes in component 2 (*mpeg1.1* and *mpeg1.2)* independent of strain [three-way ANOVA (strain, treatment, batch): treatment: *F*_(1, 23)_ = 7.660, *p* ≤ 0.011].

A PCA for the 0–6 hpf dexamethasone treatment experiment revealed only one component (Figure 2B; Supplementary Figure 1B shows the transcript abundance of individual genes). The KMO was sufficiently high (0.626) and Bartlett's test of sphericity was highly significant (Chi-square = 102.945, df = 6, *p* < 0.001), indicating that the data were adequate for a PCA. This component explained 76.6% of the variance. Loadings onto this component were: *irg1l (*0.867). *socs3a* (0.835), *mpeg1.1* (0.928), and *mpeg1.2* (0.868). Treatment with dexamethasone enhanced transcript abundance of genes independent of strain [two-way ANOVA (strain, treatment): treatment: *F*_(1, 33)_ = 6.745, *p* ≤ 0.014].

These data show that glucocorticoid treatment was effective in eliciting changes in baseline expression of (a selected set of) immune-related genes.

### Tail Fin Amputation Assay

**Figure 3:**
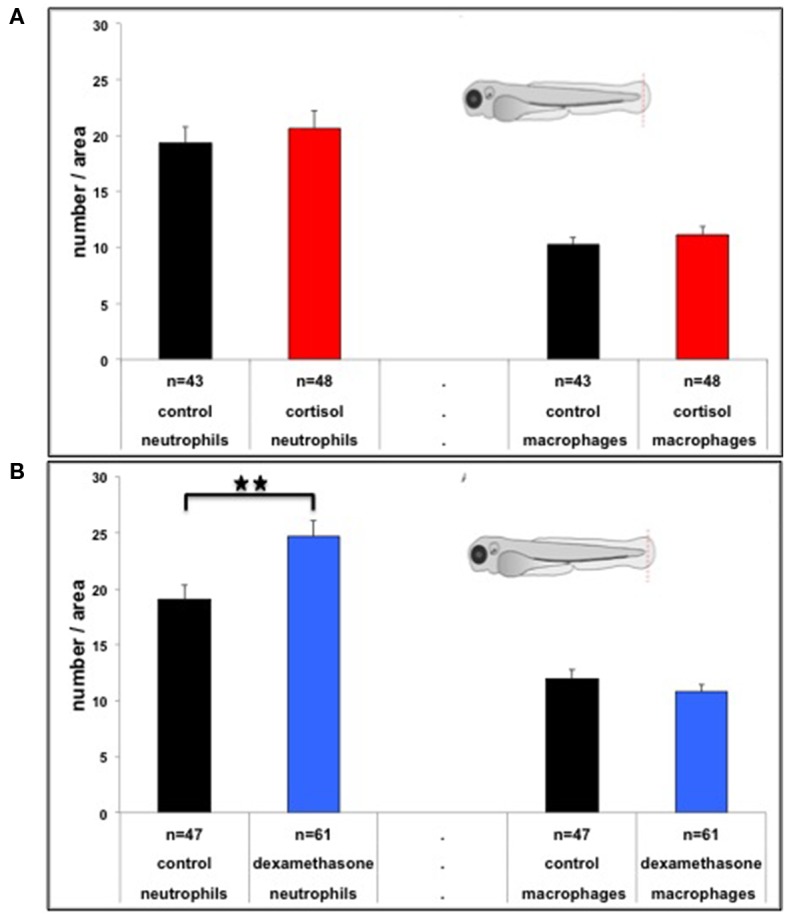
**(A)** Number of neutrophils and macrophages (mean ± SEM) at 4 hpa for the different treatments (cortisol or control). **(B)** Number of neutrophils and macrophages (mean ± SEM) at 4 hpa for the different treatments (dexamethasone or control). In both panels the picture illustrates the position of the site of the tail fin amputation in 3 dpf larvae. **p = 0.01 (Student's t-test).

The inserts in Figure 3 show the site of the tail fin amputation at 3 dpf. Neutrophil recruitment at 4 hpa was enhanced in the 0–6 hpf dexamethasone treatment group (Student's *t*-test: *t* = 2.917, df = 106, *p* ≤ 0.004; Figure 3B), but not in the 0–6 hpf cortisol treatment group (Student's *t*-test: *t* = 0.621, df = 89, ns; Figure 3A), compared to the group treated with control medium at 0–6 hpf. No effects were found for macrophage recruitment for either 0–6 hpf cortisol treatment (Student's *t*-test: *t* = 0.923, df = 89, ns; Figure 3A) or 0–6 hpf dexamethasone treatment (Student's *t*-test: *t* = −1.095, df = 106, ns; Figure 3B).

### LPS Exposure

#### Survival

In the LPS exposure experiments, we never observed any morphological changes or mortality in the 4 dpf control groups. Hence, we only present the data of the 4 dpf LPS-treated groups.

**Figure 4:**
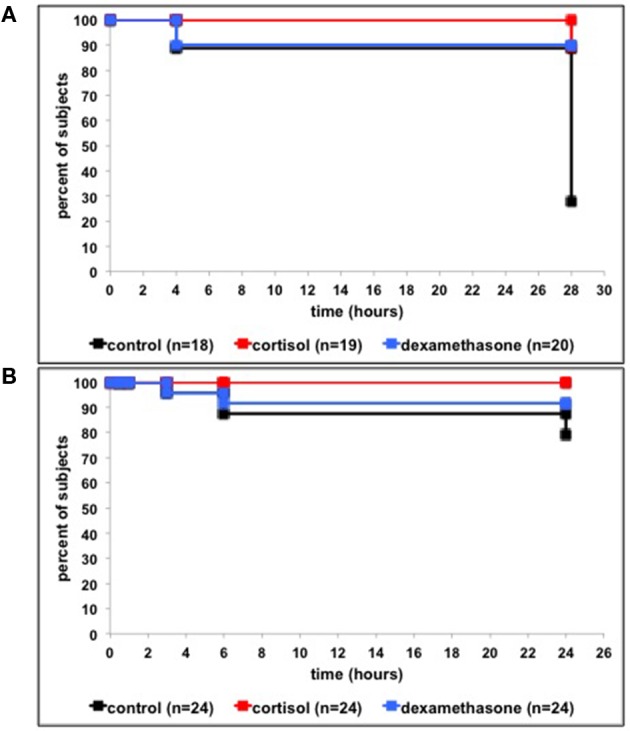
**(A)** Per cent of surviving larvae following LPS treatment (from 0 to 0.5 h) and 0–6 hpf control treatment, cortisol treatment or dexamethasone treatment in the first exposure series (time-points: 0, 4, and 28 h). **(B)** Per cent of surviving larvae following LPS treatment (from 0 to 0.5 h) and 0–6 hpf control treatment, cortisol treatment and dexamethasone treatment in second exposure series (time-points: 0, 0.5, 1, 3, 6, and 24 h).

In the first LPS exposure experiment 27.8% of the 0–6 hpf control-treated larvae survived after 28 h, while significantly higher numbers of the 0–6 hpf glucocorticoid-treated larvae survived after 28 h: cortisol: 89.5%, dexamethasone: 90.0% (Figure 4A; Log Rank Mantel Cox, Chi-square = 20.863, df = 2, *p* < 0.001). Pair-wise comparisons revealed a significant effect of cortisol vs. control, *p* < 0.001 (Log Rank Mantel Cox, Chi-square = 14.385, df = 1) and a significant effect of dexamethasone vs. control, *p* < 0.001 (Log Rank Mantel Cox; Chi-square = 12.436, df = 1). In the second LPS exposure experiment, which served as replicate for the first LPS exposure experiment, 79.2% of 0–6 hpf control-treated larvae survived after 24 h while a strong tendency for higher numbers of 0–6 hpf glucocorticoid-treated larvae survived after 24 h, in line with the first experiment: cortisol: 100.0%, dexamethasone: 91.7% (Figure 4B; Log Rank Mantel Cox, Chi-square = 5.824, df = 2, *p* ≤ 0.054). Pair-wise comparisons revealed a significant effect for cortisol vs. control, *p* ≤ 0.019 (Log Rank Mantel Cox, Chi-square = 5.466, df = 1,) but no significant effect for dexamethasone vs. control, ns (Log Rank Mantel Cox, Log Rank Mantel Cox; Chi-square = 1.370, df = 1). The latter may be related to the relatively high survival in control subjects, precluding finding a significant effect.

#### Phenotypical Effects and Gene Expression Endothelium-Related Genes

In the first LPS exposure experiment phenotypical changes were measured at 4 and 28 h, i.e., 3.5 and 27.5 h after ending exposure. Table 2 shows the numbers of dead larvae and of larvae that were alive and displayed phenotypical changes. We observed LPS-induced changes in the shape of the larvae (curved larvae), tail fin morphology (swollen or damaged tail fins) and heart cavity (oedema). Larvae were scored as either affected (at least one of these changes present) or not (no changes in any of the parameters). Supplementary Table 1 shows the scores of the individual parameters at 4 h (the number of larvae of the control treated group alive at 28 h was too low for a meaningful statistical analysis between treatments).

**Table 2:** Per cent of larvae dead, alive and affected (regardless of phenotypical changes in the tail fin, shape or cardiac area), and alive and intact at 4 and 28 h after LPS treatment (0–0.5 h) in the different treatment groups: 0–6 hpf treatment with control, cortisol-containing or dexamethasone-containing medium.

**4 h**	**Treatment**	**%Dead**	**%(alive+affected) (tail fin, shape, heart)**	**%(alive+intact)**
	Control (*n* = 18)	11.1	83.3	5.6
	Cortisol (*n* = 19)	0.0	73.7	26.3
	Dexamethasone (*n* = 20)	10.0	60.0	30.0
**28 h**	**Treatment**	**%Dead**	**%(alive+affected) (tail fin, shape, heart)**	**%(alive+intact)**
	Control (*n* = 15)	86.7	13.3	0.0
	Cortisol (*n* = 16)	12.5	37.5	50.0
	Dexamethasone (*n* = 16)	12.5	31.3	56.3

*Note that the number of larvae at 28 h is lower than at 4 h; per treatment n = 3–4 larvae (alive) were randomly selected for staining for reactive oxygen species in the tail fin (not reported here); they were not scored for phenotypical changes*.

While the scores at 4 h suggested that the LPS-induced effects were less strong in glucocorticoid-treated larvae than in control-treated larvae, this was not (as yet) significant (Chi-square = 5.81, df = 2, ns). LPS-induced effects were less strong in glucocorticoid-treated larvae than in control-treated larvae at 28 h (overall Chi-square = 25.33, df = 2, *p* < 0.001). Pair-wise comparison showed that both cortisol-treated (Chi-square = 18.05, df = 2, *p* < 0.001) and dexamethasone-treated (Chi-square = 18.34, df = 2, *p* < 0.001) groups showed fewer dead and fewer malformed larvae than control-treated subjects following LPS exposure.

**Figure 5:**
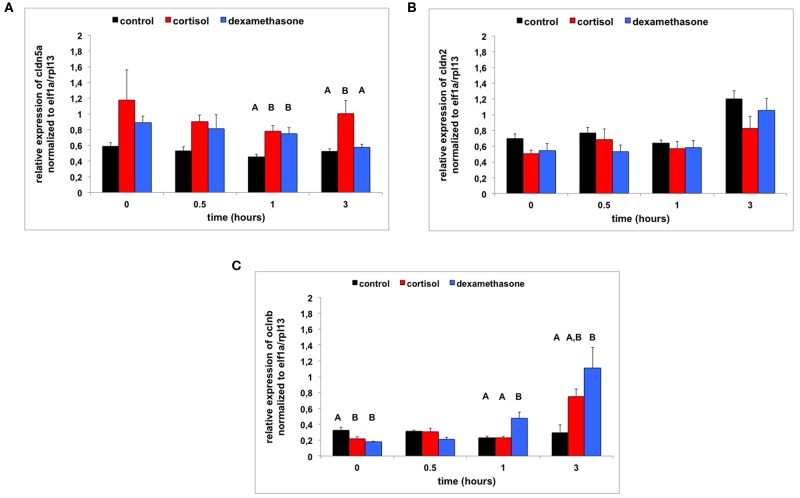
**(A)** Transcript abundance (relative normalized expression; mean+SEM) of *cldn5a* following LPS treatment (from 0 to 0.5 h) and 0–6 hpf control treatment, cortisol treatment or dexamethasone treatment (*n* = 4 samples per time-point). Groups with the same capitals do not significantly differ from one another (Tukey HSD following a significant treatment effect for this time-point). Note: control-treated subjects showed no significant change over time: *F*_(3, 12)_ = 2.009, ns. **(B)** Transcript abundance (relative normalized expression; mean + SEM) of *cldn2* following LPS treatment (from 0 to 0.5 h) and 0–6 hpf control treatment, cortisol treatment or dexamethasone treatment (*n* = 4 samples per time-point). Note: control-treated subjects showed an increased expression at 3 h: *F*_(3, 12)_ = 13.253, *p* < 0.001; Tukey HSD. **(C)** Transcript abundance (relative normalized expression; mean + SEM) of *oclnb* following LPS treatment (from 0 to 0.5 h) and 0–6 hpf control treatment, cortisol treatment or dexamethasone treatment (*n* = 4 samples per time-point). Groups with the same capitals do not significantly differ from one another (Tukey HSD following a significant treatment effect for this time-point). Note: control-treated subjects showed no significant change over time: *F*_(3, 12)_ = 0.562, ns.

In addition to the phenotypical changes that we studied, we measured expression levels of genes related to endothelial barrier function (*clnd5a, clnd2*, and *oclnb*) in the first LPS exposure experiment. Transcript abundance of *clnd5a* was higher in cortisol-treated (Tukey HSD: *p* < 0.001) and dexamethasone-treated larvae (Tukey HSD: *p* ≤ 0.067) compared to control-treated larvae [two-way ANOVA; treatment and time as independent factor; treatment: *F*_(2, 36)_ = 9.815, *p* < 0.001; Figure 5A]. Transcript abundance of *clnd2* was lower in cortisol-treated (Tukey HSD: *p* ≤ 0.034) and dexamethasone-treated larvae (Tukey HSD: *p* ≤ 0.093) compared to control-treated larvae [two-way ANOVA; treatment and time as independent factor; treatment: *F*_(2, 36)_ = 9.815, *p* < 0.001; Figure 5B]. At 3 h transcript abundance of *clnd2* was higher than at other time points in all treatment groups (Tukey HSD; *p* < 0.001; time: *F*_(2, 36)_ = 13.425, *p* < 0.001). Transcript abundance of *oclnb* was lower in cortisol-treated and dexamethasone-treated larvae than in control-treated larvae at baseline (0 h), but higher at 3 h following LPS exposure [two-way ANOVA; treatment and time as independent factor; treatment ^*^time: *F*_(3, 36)_ = 6.404, *p* < 0.001; Figure 5C].

**Figure 6:**
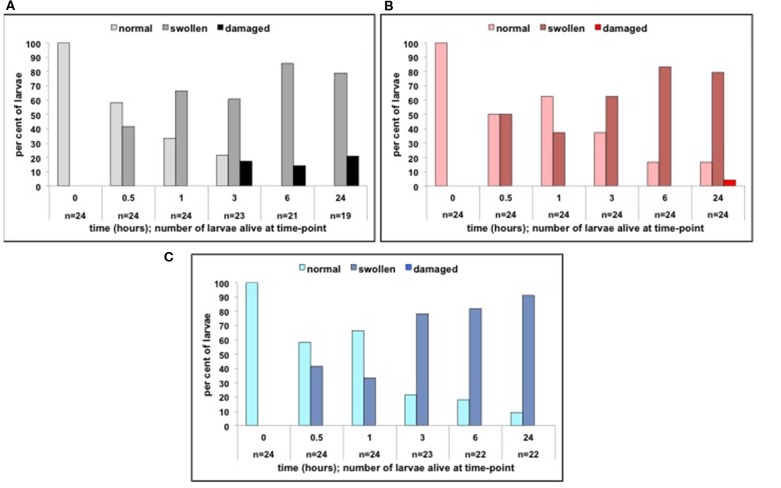
**(A)** Per cent of larvae following LPS treatment (from 0 to 0.5 h) in the 0–6 hpf control treatment group showing normal, swollen, or damaged tail fins (time-points: 0, 0.5, 1, 3, 6, and 24 h). **(B)** Per cent of larvae following LPS treatment (from 0 to 0.5 h) in the 0–6 hpf cortisol treatment group showing normal, swollen or damaged tail fins (time-points: 0, 0.5, 1, 3, 6, and 24 h). **(C)** Per cent of larvae following LPS treatment (from 0 to 0.5 h) in the 0–6 hpf dexamethasone treatment group showing normal, swollen, or damaged tail fins (time-points: 0, 0.5, 1, 3, 6, and 24 h).

In the second LPS exposure experiment we measured changes in tail fin morphology and shape as we hardly observed any oedema in the heart cavity in the first LPS exposure experiment. Figure 6 shows the changes in tail fin morphology following LPS exposure. In control-treated larvae there was a clear and rapid loss of normal tail fin structure and a shift toward swollen or damaged tail fins, while this was not the case in the glucocorticoid-treated larvae. Statistical analysis showed that at 3 h (Chi-square = 10.25, df = 4, *p* ≤ 0.036), 6 h (Chi-square = 10.27, df = 4, *p* ≤ 0.036) and 24 h (Chi-square = 10.25, df = 4, *p* ≤ 0.042) treatment groups differed significantly from one another. More in particular, both cortisol-treated larvae (3 h: Chi-square = 5.16, df = 2, *p* ≤ 0.076; 6 h: Chi-square = 6.94, df = 2, *p* ≤ 0.03; 24 h Chi-square = 5.77, df = 2, *p* ≤ 0.056) and dexamethasone-treated larvae (6 h: Chi-square = 6.98, df = 2, *p* ≤ 0.03; 24 h Chi-square = 6.53, df = 2, *p* ≤ 0.038) showed less severe changes in tail fin morphology than control-treated larvae. We did not observe a strong effect of LPS exposure on the shape of the larvae. After 24 h only small percentages of each treatment group showed a curved shape: control-treated larvae (see Supplementary Table 2 for all time points): 15.8% (*n* = 19); cortisol-treated larvae: 8.3% (*n* = 24); dexamethasone-treated larvae: 9.1% (*n* = 22); these differences were not significant (Chi-square = 1.71, df = 2, ns).

### Gene Expression Analysis

First, we explored the effects of glucocorticoid treatment through the interrelationships of transcript abundance of genes using PCA. Then in each component, we selected genes of interest of which the transcript abundance of the different treatments matched the differences in phenotypical changes and survival that we observed between treatment groups. The following genes (from the first LPS exposure experiment) were included in the analysis: *tlr2, tlr4ba, tlr4bb, tlr5a, tlr5b, il1*β, *myd88, il10, cxcr4a, cxcr4b*, and *ptpn6*.

A PCA revealed three components explaining in total 72.9% of variance (Supplementary Table 3). The KMO was sufficiently high (0.619) and Bartlett's test of sphericity was highly significant (Chi-square = 303.010, df = 55, *p* < 0.001) indicating that the data were adequate for a PCA.

**Figure 7:**
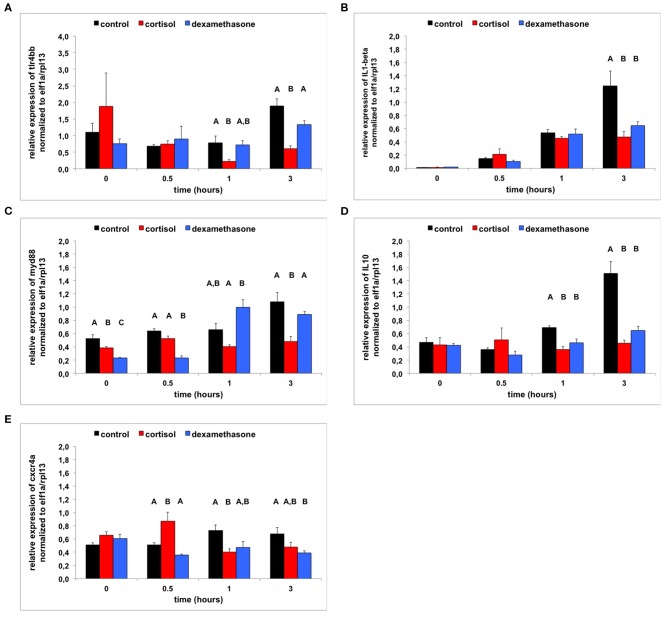
**(A)** Transcript abundance (relative normalized expression; mean+SEM) of *tlr44bb* following LPS treatment (from 0 to 0.5 h) and 0–6 hpf control treatment, cortisol treatment or dexamethasone treatment (*n* = 4 samples per time-point). Groups with the same capitals do not significantly differ from one another (Tukey HSD following a significant treatment effect for this time-point). **(B)** Transcript abundance (relative normalized expression; mean + SEM) of *IL1*β following LPS treatment (from 0 to 0.5 h) and 0–6 hpf control treatment, cortisol treatment or dexamethasone treatment (*n* = 4 samples per time-point). Groups with the same capitals do not significantly differ from one another (Tukey HSD following a significant treatment effect for this time-point). **(C)** Transcript abundance (relative normalized expression; mean + SEM) of *myd88* following LPS treatment (from 0 to 0.5 h) and 0–6 hpf control treatment, cortisol treatment or dexamethasone treatment (*n* = 4 samples per time-point). Groups with the same capitals do not significantly differ from one another (Tukey HSD following a significant treatment effect for this time-point). **(D)** Transcript abundance (relative normalized expression; mean+SEM) of *IL10* following LPS treatment (from 0 to 0.5 h) and 0–6 hpf control treatment, cortisol treatment or dexamethasone treatment (*n* = 4 samples per time-point). Groups with the same capitals do not significantly differ from one another (Tukey HSD following a significant treatment effect for this time-point). **(E)** Transcript abundance (relative normalized expression; mean+SEM) of *cxcr4a* following LPS treatment (from 0 to 0.5 h) and 0–6 hpf control treatment, cortisol treatment or dexamethasone treatment (*n* = 4 samples per time-point). Groups with the same capitals do not significantly differ from one another (Tukey HSD following a significant treatment effect for this time-point).

The first component, explaining 32.9% of the variance, consisted of the genes of different Toll-like receptors: *tlr2* (factor loading: 0.677), *tlr4ba* (0.669), *tlr4bb* (0.764), *tlr5a* (0.779), and *tlr5b* (0.838). The overall pattern of the factor regression scores across time was an inverted U-shape: a two-way ANOVA (independent factors: time and treatment) for this component revealed a highly significant effect of time [*F*_(3, 36)_ = 15.502, *p* < 0.001] with time points 0 hr and 3 h having significantly higher factor regression scores than time points 0.5 hr and 1 hr (Tukey HSD; Supplementary Table 3). Only weak effects were found between the different treatments [*F*_(2, 36)_ = 3.012, *p* ≤ 0.062; *F*_(6, 36)_ = 2.038, *p* ≤ 0.086]. The only gene of this component of which the transcript abundance of the different treatments seemed to match the differences in phenotypical changes and survival was *tlr4bb* (see Supplementary Table 4 and Figure 7A): transcript abundance increased less strongly over time in glucocorticoid-medium treated subjects than in control-medium treated subjects.

The second component, explaining 23.1% of the variance, consisted of *il1*β (factor loading: 0.940), *myd88* (0.844) and *il10* (0.758). As time progressed factor regression scores increased [two-way ANOVA (independent factors: treatment and time); time: *F*_(3, 36)_ = 68.309, *p* < 0.001] with a *post-hoc* Tukey HSD revealing that the factor regression scores differed significantly from one another at all-time points (Supplementary Table 3). The 0–6 hpf control group had increasingly higher factor regression scores than the 0–6 hpf cortisol treated group and the 0–6 hpf dexamethasone treated group as time progressed [Supplementary Table 3; Tukey HSD; treatment: *F*_(2, 36)_ = 22.664, *p* < 0.001; treatment^*^time: *F*_(6, 36)_ = 13.491, *p* < 0.001]. Of all genes of this component, transcript abundance of the different treatments seemed to match the differences in phenotypical changes and survival (Supplementary Table 4 and Figures 7B–D): transcript abundance increased less strongly over time in glucocorticoid-medium treated subjects than in control-medium treated subjects.

The third component, explaining 16.9% of the variance, consisted of regulators of the immune response: *cxcr4a* (factor loading: 0.794), *cxcr4b* (0.809) and *ptpn6* (0.799). Overall the factor regression scores decreased over time: a two-way ANOVA (independent factors: treatment and time) revealed a highly significant effect of time [*F*_(3, 36)_ = 4.464, *p* ≤ 0.009] with 3 and 0 h being significantly different from one another (Tukey HSD). The 0–6 hpf dexamethasone treated group had lower factor regression scores than the 0–6 hpf control treated group and the 0–6 hpf cortisol treated group [treatment: *F*_(2, 36)_ = 11.056, *p* < 0.001; treatment^*^time: *F*_(6, 36)_ = 5.571, *p* < 0.001). The only gene of this component of which the transcript abundance of the different treatments seemed to match the differences in phenotypical changes and survival was *cxcr4a* (see Supplementary Table 4 and Figure 7E): transcript abundance increased in control-medium treated subjects but decreased in glucocorticoid-medium treated subjects.

## Discussion

The data of this study showed that treatment of zebrafish embryos with cortisol or dexamethasone during the first 6 h after fertilization modulated the function of the immune system and thereby enhanced survival after an immune challenge. This suggests that in zebrafish maternal stress through enhancing oocyte cortisol levels and thereby increased GR stimulation leads to an adaptive response to immune challenges.

### Baseline Expression of Immune-Related Genes

Hartig et al. (21) have shown that 5-day exposure to (1 μM) cortisol in zebrafish embryos/larvae (0–5 dpf) enhanced baseline expression of immune-related genes such as of *socs3a, mpeg1*, and *irg1l* at 5 dpf. Here, we show that exposure at the first 6 h of life (0–6 hpf) is already sufficient to induce enhanced baseline expression of these genes at 5 dpf. As GR is the only corticosteroid receptor present in these early life stages (3, 7, 8), this suggests that activation of GR in these early stages is responsible for mediating these effects. Indeed, we show that 0–6 hpf exposure to the specific GR agonist dexamethasone enhanced baseline expression of these immune-related genes in 5 dpf larvae as well. This is in general agreement with data from studies showing that exposure of embryos/larvae to other GR agonists (for variable time-periods from fertilization) increased baseline expression of (some of) these genes (42–44).

We have previously shown that 0–6 hpf exposure to cortisol-containing medium enhanced baseline levels of cortisol compared to 0–6 hpf exposure to control medium at 5 dpf in larvae of the AB strain, but not in larvae of the TL strain (10). In addition, we have previously shown that 0–6 hpf exposure to dexamethasone-containing medium did not change baseline levels of cortisol compared to 0–6 hpf exposure to control-medium at 5 dpf in larvae of the AB or TL strain (36). Overall these data therefore strongly suggest that the effects on the expression of immune-related genes in AB and TL larvae are not related to changes in baseline levels of cortisol.

While we have previously observed substantial differences between larvae of the AB and TL strains in gene expression, physiology and behavior (10, 22–24), the effects of 0–6 hpf exposure to cortisol and dexamethasone on baseline expression of immune-related genes were similar in both strains. This suggests a robust strain-independent effect.

### Tail Fin Amputation Assay

It has been shown that tail fin amputation in 3 dpf larvae leads to a rapid recruitment of macrophages (within 2 hpa) remaining at a plateau for at least 24 h thereafter, while the number of neutrophils reaches a peak 4 hpa declining thereafter (30). Here, we observed that dexamethasone increased the number of neutrophils at 4 hpa, while it had no effect on the number of macrophages at 4 hpa. These data suggest that pre-exposed subjects mount a stronger response to injury, possibly enhancing protection against invading micro-organisms (26).

Dexamethasone, but not cortisol, exposure was effective in increasing the recruitment of neutrophils. Two explanations may be forwarded for this. First, this may be related to dexamethasone's higher affinity for GR (25). In line with this we observed enhanced expression of FK506 binding protein 5 (*fkbp5*), a marker for levels of GR stimulation [e.g., (42, 43)] in dexamethasone-exposed embryos, but not cortisol-exposed embryos (10, 36). Second, it has been shown neutrophil recruitment, but not macrophage recruitment, is sensitive to acute treatment with GR agonists, including cortisol, which decreases the number of neutrophils at 4 hpa (26, 30, 31). We have observed that baseline levels of cortisol are enhanced following cortisol but not dexamethasone treatment (10, 36). Hence, in the case of cortisol pre-exposure enhancement of neutrophil recruitment through GR stimulation (as indicated by the effect of dexamethasone) may potentially be offset at 3 dpf by the higher baseline levels of cortisol. If so, this suggests a fine-tuning of the response. Future studies should address these alternative explanations.

### LPS Challenge

In line with results from other studies (32–35) LPS exposure produced phenotypical changes (tail fin oedema, swollen or damaged tail fins, and curved animals), mortality and increased expression levels of immune-related genes. To assess the molecular mechanisms underlying the effects of LPS exposure, we measured the expression of *cldn5a, cldn2*, and *oclnb*; genes of which the expression has been shown to be changed following LPS exposure (33, 35) and which are involved in endothelial barrier function (45–48). The strong increase in the expression of *cldn2* at 3 h in control-treated subjects is in line with data from other studies (33, 35). Increased expression of *cldn2* is associated with endothelial hyper-permeability due to increased pore-pathway activity possibly mediated by increased expression of IL-13 (45–48). At variance with other studies (33, 35) we did not observe strongly decreased expressions of *cldn5a* and *oclnb*. Decreased expression levels of *cldn5a* and *oclnb* are related to endothelial hyper-permeability due a lower sealing function of the pore-pathway and a less functional leaky pathway, respectively (45–48). One reason for this may be that we measured gene expressions at 3 h after the start of the LPS exposure, while in other studies this was measured at substantially later time points, i.e., 6 and 8 h (33, 35).

In both LPS exposure experiments both cortisol and dexamethasone exposure at 0–6 hpf were associated with milder effects to LPS compared to control exposure at 0–6 hpf, indicating a robust effect of glucocorticoid exposure. This was expressed as milder phenotypical changes (lower number of larvae expressing tail fin oedema, damaged tail fins or curved animals), lower mortality, and lower gene expression of immune-related genes, such as *il1*β. As to the expression of endothelium-related genes we noted that the expression of *cldn5a* was higher and expression of *cldn2* lower in cortisol- and dexamethasone-treated subjects compared to control-treated subjects. This suggests a lower permeability of the endothelium due to a lower pore-pathway activity (45–48) supporting the milder phenotypical effects following LPS exposure. The expression of *oclnb* was strongly increased after 3 h in 0–6 hpf cortisol-treated and dexamethasone-treated subjects compared to control-treated subjects. This suggests lower permeability due to more protective leaky pathway activity (45–48). It has been shown that 24 h following LPS challenge in zebrafish larvae the expression of *oclnb* is strongly up-regulated facilitating tissue-repair (33). Again, the data support the milder phenotypical effects that we see following LPS exposure in cortisol-treated and dexamethasone-treated subjects compared to control-treated subjects.

To explore the underlying mechanisms of glucocorticoid treatment we measured the expression of a series of genes of interest and related their expression to the outcome of the phenotypical changes and mortality. Five genes stood out in this respect, reflecting two receptors (*tlr4bb* and *cxcr4a)*, a factor involved in the transduction pathway of the expression of cytokines (*myd88*) and two cytokines (*il1*β and *il10*).

While LPS exerts its effects through transduction mechanisms following binding to TLR4 in mammals (45, 49), in zebrafish this is not clear as yet: *tlr4ba* and *tlr4bb* have been suggested to be paralogues rather than homologs and TLR4BA and TLR4BB have thus far not been shown to be activated by LPS possibly by lack of a binding site for LPS (50, 51). However, TLR4BB has been shown to be involved in inflammatory processes as *tlr4bb* transcript abundance is increased following tail fin amputation (30). Here, we observed an increase in *tlr4bb* expression in 0–6 hpf control-treated subjects over time, accompanied by a strong inflammatory response, which was less strong in 0–6 hpf cortisol-treated or dexamethasone-treated subjects, accompanied by a milder inflammatory response. This suggests a role for TLR4BB in the LPS-induced response.

In humans CXCR4 has been implicated in recognition of LPS or being part of a “LPS sensing apparatus” in addition to TLR4 (52, 53). Furthermore, LPS increases the expression of *cxcr4* through an NF-κB signaling pathway associated with increased micro-vascular leakage in the lungs (54) or increased colorectal tumor metastasis (55). Here, we observed an increase in *cxcr4a* expression in 0–6 hpf control-treated subjects over time, which was absent in 0–6 hpf cortisol-treated or dexamethasone-treated subjects. This difference in gene expression may be associated with differences in the extent of vascular leakage as suggested by the differences in the expression of genes involved in the endothelial barrier and differences in tail fin oedema between treatments as discussed above. In zebrafish *cxcr4a* is found in endothelial cells (blood vessels), while *cxcr4b* is not (56), which may explain that we only observed a phenotype-related effect for the expression profile of *cxcr4a*. Interestingly, CXCR4 has been implicated in the development of tolerance to lethal doses of LPS in zebrafish larvae (32, 34). Thus, this suggests that CXCR4 is involved in modulating the response to LPS.

Overall therefore our data warrant further studies into the role of TLR4BB and CXCR4 in LPS-induced sepsis in zebrafish as well as into the effects of early life glucocorticoid stimulation hereon.

Earlier studies have shown that MYD88 knockout larvae show enhanced survival to LPS challenge (33) and no increase in *il1*β expression (57). Lethal, but not sub-lethal, doses of LPS have been found to be associated with high expression levels of *il1*β and *il10* in zebrafish larvae, indicative of a hyper-inflammatory response (32). In line with these findings we observed that the expression levels of *myd88, il1*β, and *il10* were strongly increased 3 h after LPS challenge associated with low survival in 0–6 hpf control-treated subjects, but lower levels of expression of all three genes with higher survival in 0–6 hpf cortisol-treated and dexamethasone-treated subjects. Myd88 is an adaptor protein critical to toll-like receptor signaling [except for TLR3; (49)] and IL1β receptor signaling [see (38, 57)] and thereby cytokine expression. It should be noted that *myd88* expression was already low in 0–6 hpf cortisol-treated and dexamethasone-treated subjects, suggesting lower transduction pathway activity, possibly leading to a lower stimulation of inflammatory pathways. It is clear that this deserves further study.

As indicated above following cortisol or dexamethasone treatment at 0–6 hpf we noted increased base-line expression levels of *irg1l, socs3a, mpeg1.1*, and *mpeg1.2* compared to control treatment at 0–6 hpf. These increased base-line levels may aid in increased clearance of bacteria and preventing excessive inflammation and hence aid in increasing survival (26, 58, 59).

The data of LPS exposure in larvae observed here seem to match the data of LPS exposure in adult zebrafish following 0–5 dpf exposure to cortisol: LPS exposure did not increase *il1*β expression in different tissues measured (21). Unfortunately no survival was measured in the latter study.

Overall these data suggest that the response to LPS of subjects pre-exposed to cortisol or dexamethasone is less strong than the response of subjects of pre-exposed to control medium. How differences in survival, phenotype and gene-expression levels are causally related remains to be studied. As we have observed in larvae of the AB strain that 0–6 hpf exposure to cortisol increased baseline levels of cortisol at 5 dpf, while 0–6 hpf exposure to dexamethasone did not change baseline levels of cortisol at 5 dpf (10, 36), the data suggest that these effects are not related to changes in baseline levels of cortisol.

### Limitations

A clear limitation is that we only used one dose of cortisol and dexamethasone. For convenience we used equimolar doses of cortisol and dexamethasone, which may have led to different levels of stimulation of GR 0–6 hpf. Thus, in future studies different dose-ranges may be warranted, e.g., to study whether higher concentrations of cortisol in the tail fin amputation assay have an effect on neutrophil recruitment, and to assess whether higher doses have similar protective effects in the LPS-exposure paradigm.

Regarding the tail fin amputation assay it has been shown that glucocorticoids may play a role in the differentiation of macrophages into a pro-inflammatory (M1) phenotype (31). So, future studies should study in more detail the effects of early life exposure of glucocorticoids on the inflammatory response and wound healing. Similarly, we used LPS to induce a hyper-inflammatory response, i.e. sepsis (33, 35), as a model to study the effectiveness of our early life treatments. To assess the ecological relevance of our findings and their more general nature the effects of early-life exposure on larval exposure to different pathogens, such as of bacterial, viral or fungal origin, should be studied [see e.g., (12, 57)].

While the effects of 0–6 hpf glucocorticoid treatment appeared to be similar in both LPS exposure experiments, the effects of 0–6 hpf control treatment seemed to differ, i.e., in the second experiment more subjects survived than in the first experiment. This highlights the value of and need for biological replication. We predict therefore that this would also be reflected in the expression of endothelium-related and immune-related genes in control subjects in the second experiment, i.e., the differences between control treatment and glucocorticoid treatment would be less strong. In future experiments therefore we will routinely analyse a number of key genes alongside phenotypical changes. As to the observation that control-treated larvae showed variable responses, this is not uncommon as we also observed variable responses in other immune-related paradigms such as the response to a dextran sodium sulfate (DSS) challenge (Van den Bos et al., unpublished observations). While as yet speculative differences in baseline levels of expression of *il1*β may be one associated factor as we observed that higher baseline levels seem associated with milder responses to LPS (Van den Bos et al., unpublished data). This is not unprecedented as this has also been observed in mice: enhanced levels of IL1 are associated with a milder response to LPS (60). It has been shown that following hatching *il1*β expression increases due to exposure to microbes in the medium (61). This tunes the activity of the innate immune system of the zebrafish larvae and determines thereby their disease resistance. In the laboratory however this may lead to variation between experimental series as the microbial load of the medium may vary from experimental series to series. It is clear that this warrants further studies. It has been suggested that tuning the innate immune system occurs through chromatin modifications [see e.g., (61–64)]. Our future studies are directed at understanding the variable responses in this context. In addition our future studies are directed at understanding how 0–6 hpf glucocorticoid treatment affects this as we do not observe such variation following this treatment.

The immune system affects HPI-axis activity and *vice versa* (1). However, we did not address changes in HPI-axis activity as a consequence of our procedures (tail fin amputation or LPS exposure) in this study as the primary aim was to study whether our pre-treatments would affect immune function. Still changes in HPI-axis activity may be anticipated. For example, a recent study showed in European sea bass (*Dicentrarchus labrax*) larvae 5 day post-hatching that at 120 h following infection with the bacterium *Vibrio anguillarum*, when mortality was already high, HPI-axis activity increased (65). This increased activity coincided with increased expression of pro-inflammatory and anti-inflammatory genes. While the underlying mechanism was not clear as yet, the data show that infections may impact HPI-axis activity. It has been shown that early-life exposure to cortisol dampens the response of the HPI-axis to stressors in zebrafish larvae (15). Hence, future studies should address how early-life exposure to cortisol affects the relationship between immune function and HPI-axis activity, and how this relates to the increased survival that we have observed here.

We have used 0–6 hpf cortisol exposure by the medium as a model or proxy of increased oocyte levels of cortisol due to chronic stress in mothers (10), while others have used micro-injection of cortisol in the yolk of single-cell stage embryos (13–15). Chronic stress, whether due to excessive predation, food shortage, crowding or out-of-range-temperatures, is associated with increased levels of cortisol (1) and at this level these procedures may be a valid approach of mimicking maternal stress. Still, these different stressors may have additional neuro-endocrine and/or metabolic signatures that affect oocyte yolk-sac contents and hence thereby development of embryos and larvae. Thus, future experiments should compare the present data with data from chronically stressed mothers using different types of stressors. In addition, future experiments should assess whether a paternal contribution exists by subjecting fathers to chronic stress.

## Conclusion

These data show that early-life exposure to cortisol, as a model or proxy of maternal stress, induces an adaptive response to immune challenges, which seems mediated via the glucocorticoid receptor.

These data are of relevance for both ecological research (66) and biomedical research (18) in understanding the effects of stressful conditions and exposure to endocrine disruptors on disease susceptibility and survival of offspring. Future studies should elucidate whether the effects also hold in subjects of other strains than AB revealing thereby fundamental aspects of how early-life levels of cortisol affects offspring functioning and survival.

## Data Availability Statement

The datasets generated for this study are available on request to the corresponding author.

## Ethics Statement

The experiments were carried out on zebrafish embryos and larvae (0–5 dpf). Hence, we did not need a license or review of a committee. It should be noted that all experiments were carried out in accordance with the Dutch Experiments on Animals Act (http://wetten.overheid.nl/BWBR0003081/2014-12-18), the European guidelines for animal experiments (http://Directive2010/63/EU; http://eur-lex.europa.eu/legal-content/NL/TXT/HTML/?uri=CELEX:32010L0063) and institutional regulations (Radboud University or Leiden University).

## Author Contributions

RB and MS conceived the project. SC, KT, JA, and RW performed experiments. RB, SC, KT, JA, and RW analyzed data. JZ and SC performed qPCR analysis. RB, GF, and MS wrote the manuscript.

## Conflict of Interest

The authors declare that the research was conducted in the absence of any commercial or financial relationships that could be construed as a potential conflict of interest.

## Supplementary Material

Supplementary Figure 1 **(A)**Transcript abundance (relative normalized expression; mean + SEM) of the different immune related genes for the different treatments (cortisol or control) and strains (AB or TL). One subject was removed from the statistical analyses (TL control) as it was a consistent outlier following Grubb's outlier test. Treatment effects were found for *irg1l* [*F*_(1, 23)_ = 11.789, *p* ≤ 0.01; treatment ^*^ strain: *F*_(1, 23)_ = 8.446, *p* ≤ 0.01; AB: *p* ≤ 0.05; TL: *p* ≤ 0.01] and mpeg1.1 [*F*_(1, 23)_ = 8.614, *p* ≤ 0.01]. **(B)** Transcript abundance (relative normalized expression; mean+SEM) of the different immune related genes for the different treatments (dexamethasone or control) and strains (AB or TL). Treatment effects were found for *irg1l* [*F*_(1, 33)_ = 6.484, *p* ≤ 0.05], *socs3a* [*F*_(1, 33)_ = 7.655, *p* ≤ 0.01] and mpeg1.1 [*F*_(1, 33)_ = 5.487, *p* ≤ 0.05].

Supplementary Table 1 Per cent of affected larvae at 4hrs following LPS exposure (0–0.5 h) for the different treatment groups: 0–6 hpf treatment with cortisol, dexamethasone or control medium.

Supplementary Table 2 Per cent of larvae, which are curved following LPS exposure (0–0.5 h), in the different treatment groups: 0–6 hpf treatment with cortisol-containing medium, dexamethasone-containing medium, or control medium.

Supplementary Table 3 Mean (±SEM) factor regression scores for the different treatments of the three components that emerged from the PCA (see text for details).

Supplementary Table 4 Mean (±SEM) transcript abundance of different genes involved in the inflammatory response (*n* = 4 per time point). Numbers before genes indicate to which component of the PCA genes belong (see Supplementary Table 3 and text).

## Abbreviations

ANOVA: analysis of variance
dpf: days post-fertilization
GFP: green fluorescent protein
GR: glucocorticoid receptor
hpa: hours post-amputation
hpf: hours post-fertilization
HPI-axis: hypothalamus - pituitary - interrenal axis
IL: interleukine
KMO: Kaiser-Meyer-Olkin
LPS: lipopolysaccharide
MANOVA: multivariate analysis of variance
MR: mineralocorticoid receptor
ns: not significant
PBS: phosphate buffered saline
qPCR: quantitative polymerase chain reaction
SEM: standard error of the mean
TLR: Toll-like receptor
Tukey HSD: Tukey Honestly Significant Difference.
