# Supplementary figures and images for: Early Life Glucocorticoid Exposure Modulates Immune Function in Zebrafish (Danio rerio) Larvae
Source: Front Immunol. 2020 Apr 29;11:727. doi: 10.3389/fimmu.2020.00727 (PMC7201046; doi:10.3389/fimmu.2020.00727)

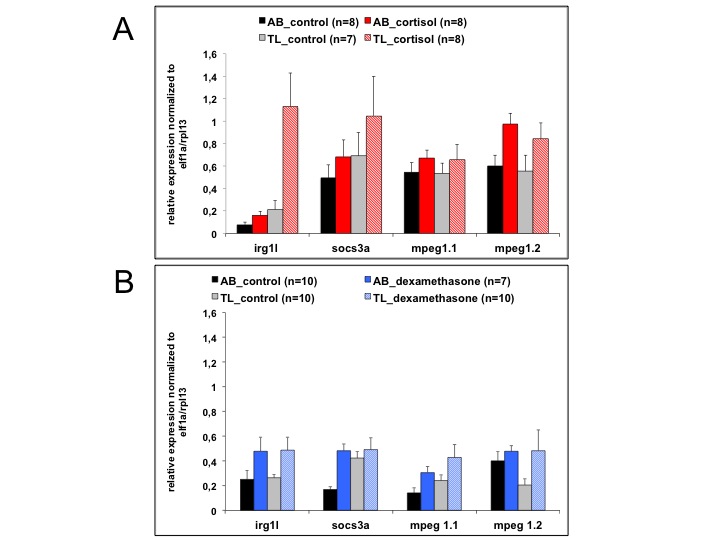

Supplement: Supplementary Figure 1 — (A)Transcript abundance (relative normalized expression; mean + SEM) of the different immune related genes for the different treatments (cortisol or control) and strains (AB or TL). One subject was removed from the statistical analyses (TL control) as it was a consistent outlier following Grubb's outlier test. Treatment effects were found for irg1l [F(1, 23) = 11.789, p ≤ 0.01; treatment * strain: F(1, 23) = 8.446, p ≤ 0.01; AB: p ≤ 0.05; TL: p ≤ 0.01] and mpeg1.1 [F(1, 23) = 8.614, p ≤ 0.01]. (B) Transcript abundance (relative normalized expression; mean+SEM) of the different immune related genes for the different treatments (dexamethasone or control) and strains (AB or TL). Treatment effects were found for irg1l [F(1, 33) = 6.484, p ≤ 0.05], socs3a [F(1, 33) = 7.655, p ≤ 0.01] and mpeg1.1 [F(1, 33) = 5.487, p ≤ 0.05]. [file Image_1.JPEG]
