# Supplementary material for: Early Life Glucocorticoid Exposure Modulates Immune Function in Zebrafish (Danio rerio) Larvae
Source: Front Immunol. 2020 Apr 29;11:727. doi: 10.3389/fimmu.2020.00727 (PMC7201046; doi:10.3389/fimmu.2020.00727)
Supplement: Supplementary Table 1 — Per cent of affected larvae at 4hrs following LPS exposure (0–0.5 h) for the different treatment groups: 0–6 hpf treatment with cortisol, dexamethasone or control medium. [file Table_1.docx]

| **Treatment** | **shape** |  | **tail fin** |  | **heart** |
| --- | --- | --- | --- | --- | --- |
|  | **%curved** | **%normal** | **%swollen** | **%damaged** | **%oedema** |
| **control (n=16)** | 50.0 | 18.8 | 75.0 | 6.3 | 6.3 |
| **cortisol (n=19)** | 10.5 | 31.6 | 68.4 | 0.0 | 5.3 |
| **dexamethasone (n=18)** | 11.1 | 33.3 | 61.1 | 5.6 | 5.6 |
|  |  |  |  |  |  |
| **Chi-square** | 9.8, df=2, p≤0.0074 |  | 2.12, df=4, ns |  | 0.02, df=2, ns |

**Supplementary table 1**: Per cent of affected larvae at 4hrs following LPS exposure (0-0.5hr) for the different treatment groups: 0-6 hpf treatment with cortisol, dexamethasone or control medium. Shown are shape: curved; tail fin: swollen or damaged; heart: oedema. In the lower row overall statistics are indicated. It should be noted that both in the cortisol-treated (Chi-square=4.84, df=2, p≤0.03) and dexamethasone-treated (Chi-square=4.44, df=2, p≤0.04) groups fewer curved larvae were present than in the control-treated larvae.
