# Supplementary material for: Early Life Glucocorticoid Exposure Modulates Immune Function in Zebrafish (Danio rerio) Larvae
Source: Front Immunol. 2020 Apr 29;11:727. doi: 10.3389/fimmu.2020.00727 (PMC7201046; doi:10.3389/fimmu.2020.00727)
Supplement: Supplementary Table 2 — Per cent of larvae, which are curved following LPS exposure (0–0.5 h), in the different treatment groups: 0–6 hpf treatment with cortisol-containing medium, dexamethasone-containing medium, or control medium. [file Table_2.DOCX]

| **treatment** | **0hr** | **0.5hr** | **1hr** | **3hrs** | **6hrs** | **24hrs** |
| --- | --- | --- | --- | --- | --- | --- |
| **control** | 0 (24) | 0 (24) | 12.5 (24) | 0 (23) | 0 (21) | 15.8 (19) |
| **cortisol** | 0 (24) | 0 (24) | 0 (24) | 0 (24) | 0 (24) | 8.3 (24) |
| **dexamethasone** | 0 (24) | 0 (24) | 4.2 (24) | 4.3 (23) | 9.1 (22) | 9.1 (22) |

**Supplementary table 2**: Per cent of larvae, which are curved following LPS exposure (0-0.5hr), in the different treatment groups: 0-6 hpf treatment with cortisol-containing medium, dexamethasone-containing medium or control medium. Between brackets: the number of subjects at each time-point; these numbers vary over time as subjects die because of the LPS treatment.
