# Supplementary material for: Early Life Glucocorticoid Exposure Modulates Immune Function in Zebrafish (Danio rerio) Larvae
Source: Front Immunol. 2020 Apr 29;11:727. doi: 10.3389/fimmu.2020.00727 (PMC7201046; doi:10.3389/fimmu.2020.00727)
Supplement: Supplementary Table 3 — Mean (±SEM) factor regression scores for the different treatments of the three components that emerged from the PCA (see text for details). [file Table_3.DOCX]

| **Time (hrs)** | **component**  **1** |  |  | **component**  **2** |  |  | **component**  **3** |  |  |
| --- | --- | --- | --- | --- | --- | --- | --- | --- | --- |
|  | **control** | **cortisol** | **dex** | **control** | **cortisol** | **dex** | **control** | **cortisol** | **dex** |
| **0** | +0.33±0.24 | +0.89±0.82 | +0.96±0.28 | -0.90±0.06 | -0.97±0.18 | -1.01±0.08 | +0.26±0.11^A,B^ | +0.94±0.27^A^ | -0.18±0.08^B^ |
| **0.5** | -0.85±0.07 | -0.17±0.39 | -0.41±0.49 | -0.23±0.07^A^ | -0.04±0.20^A^ | -0.85±0.10^B^ | -0.38±0.17^A^ | +1.78±0.48^B^ | -1.04±0.32^A^ |
| **1** | -0.93±0.05 | -1.04±0.22 | -0.55±0.26 | +0.64±0.10^A^ | -0.28±0.08^B^ | +0.60±0.16^A^ | +1.02±0.28 | -0.15±0.41 | -0.47±0.48 |
| **3** | +0.76±0.5^A^ | -0.35±0.07^B^ | +1.37±0.25^C^ | +2.43±0.41^A^ | +0.10±0.26^B^ | +0.49±0.10^B^ | -0.49±0.40 | -0.57±0.38 | -0.72±0.36 |

**Supplementary Table 3**: Mean (±SEM) factor regression scores for the different treatments of the 3 components that emerged from the PCA (see text for details). dex = dexamethasone. Super-scripts: different capitals indicate significant differences between groups (Tukey HSD) following a significant treatment effect at that particular time-point.
